# Knowledge translation in health research: A novel approach to health sciences education

**DOI:** 10.3885/meo.2009.T0000142

**Published:** 2009-08-18

**Authors:** Sylvia Reitmanova

**Affiliations:** Division of Community Health & Humanities, Memorial University of Newfoundland, St. John's, NL, Canada

**Keywords:** health sciences curriculum, knowledge translation, research utilization

## Abstract

The salient role of knowledge translation process, by which knowledge is put into practice, is increasingly recognized by various research stakeholders. However, medical schools are slow in providing medical students and health professionals engaged in research with the sufficient opportunities to examine more closely the facilitators and barriers to utilization of research evidence in policymaking and implementation, or the effectiveness of their research communication strategies. Memorial University of Newfoundland now offers a knowledge translation course that equips students of community health and applied health research with the knowledge and skills necessary for conducting research, that responds more closely to the needs of their communities, and for improving the utilization of their research by a variety of research consumers. This case study illustrates how the positive research outcomes resulted from implementing the knowledge translation strategies learned in the course. Knowledge translation can be useful also in attracting more funding and support from research agencies, industry, government agencies and the public. These reasons offer a compelling rationale for the standard inclusion of knowledge translation courses in health sciences education.

## Methods

In this paper, I reflect on the knowledge translation (KT) theory that I was fortunate to explore in an elective course during my graduate program in community health at Memorial University of Newfoundland. My aim is to illustrate that including a KT course in health sciences curricula can equip health researchers with the knowledge and skills necessary for improving their research's utilization by policymakers and service providers. The steps outlined in the following paragraphs can serve as a useful learning guide to medical students and health professionals conducting health research who would like to increase the utilization of their research findings. In the following paragraphs, I will examine the KT theories and strategies learned in my elective course and illustrate them with concrete practical examples to document how the positive research outcomes resulted from practical application of the learned course material.

Currently, all medical schools across North America offer a variety of core courses about quantitative and/or qualitative research theories and methods, to enable students to develop solid research skills necessary for conducting sound, objective, and ethical health research. Applying the acquired knowledge, students, as well as other researchers often expect that the research they produce as a result of their hard work and dedication will be taken seriously by those who develop policies, provide services, or implement new technologies. Lomas drew a compelling picture when he equated these expectations with the image of a retail store in which “researchers are busy filling shelves of a shop-front with a comprehensive set of all possible relevant studies that a decision maker might some day drop by to purchase.”^1^ Nonetheless, it is well-documented that evidence-based health research *per se* currently does not have a large direct influence on policymakers, administrators, or clinicians, due to a variety of political, organizational, procedural, financial and other factors.^1^,^2^ Successful research implementation depends on two other factors: “the context in which the proposed change is to be implemented, and the mechanisms by which the change is facilitated.”^3^ It means that even low research evidence (evidence not based on randomized control trials, systematic reviews, high levels of consensus among experts, and patients’ involvement) may inform policymaking and implementation if the facilitation process is intensive and the environment receiving the research outcomes is conducive to change.^3^

Experts in the field of knowledge application developed a number of quality theories, methods, and strategies for overcoming the existing silos between researchers and research consumers. However, the core research methods courses currently taking place in medical schools rarely offer to students engaged in research an opportunity to explore the process of KT and research utilization, which would allow them to examine the role of research evidence in decision making, to identify the factors that behave as facilitators or barriers to utilization of research evidence in policymaking and implementation, or to investigate the effectiveness of research communication strategies.

It must be noted here, however, that no single definition of KT exists in literature. In fact, Graham's team found 11,800 Google search hits for the term KT in 2006.^4^ Even more hits were generated by related terms such as *knowledge transfer*, *knowledge exchange* and *research utilization*. Despite the large number of hits, the authors were able to select the two most prominent definitions of KT. The first definition, coined by Canadian Institutes of Health Research (CIHR), describes KT as “the exchange, synthesis and ethically-sound application of knowledge – within a complex system of interactions among researchers and users – to accelerate the capture of the benefits of research for Canadians through improved health, more effective services and products, and a strengthened health care system.”^4^ The second definition, adopted by the US National Center for the Dissemination of Disability Research, frames KT as “the collaborative and systematic review, assessment, identification, aggregation and practical application of high-quality disability and rehabilitation research by key stakeholders (i.e., consumers, researchers, practitioners, policy makers) for the purpose of improving the lives of individuals with disabilities.”^4^ Although these two definitions explain KT in the context of health research, KT continues to gain strong currency in a variety of other disciplines such as environmental engineering,^5^ forest industry^6^ and children's poverty,^7^ to name just a few. Regardless of the application field, the process by which knowledge is translated into practice can be organized around two main phases (pre-research environmental assessment, linkage and exchange; and post-research communication) which I will define and illustrate with practical examples in the following section.

**Practical application of KT theory in health research** - This section will outline specific KT strategies and steps, recommended by available literature sources about KT theories, which I applied during the pre- and post-research phase of my study.

**Environmental assessment** - During my graduate research project, I intended to examine local immigrants’ mental health determinants and the barriers they encountered in the utilization of available mental healthcare services. Thanks to my immigrant experiences and medical training in psychiatry, I was aware that developing accessible support mechanisms and services responsive to immigrants’ needs was essential to mitigating the negative effects of immigration-related difficulties caused by excessive social stress, marginalization, social isolation, cultural conflicts, low socioeconomic status, and racial discrimination, all of which make immigrants vulnerable to mental illness.^8^ However, during my KT class, I learned that in order to facilitate the uptake of my research findings and recommendations by local policymakers and service providers, first I had to determine if researching the issues of immigrant mental health and well-being was a relevant and timely issue in the local context. Therefore, I had to conduct an environmental assessment, defined in literature as “scanning the broad political, social and economic environments to determine current trends and gaps in the research.”^9^ In addition to review panels and outreach programs, this scan can include a literature review, an Internet search, and consultation with the research-relevant audiences. I implemented the last three strategies in my environmental assessment.

The literature review of the materials relevant to the mental health of local immigrants showed that, in fact, there was a big research gap in this particular area. Very little information was available regarding immigrants’ mental health status^10^ and none on the determinants of their health or barriers to their care. With respect to mental health services in Newfoundland and Labrador (NL) that could respond to the unique needs of local immigrants as a culturally and linguistically diverse population, no plan to develop such services was present in the newly designed provincial mental health plan.^11^

The Internet search further enabled me to identify possible research audiences who, in addition to immigrants, included local policymakers, service providers, and researchers in the area of mental health and immigration. These include the NL Psychiatric Association, the Association of Psychologists in NL, the local branch of the Canadian Mental Health Association (CMHA), Health and Community Services-St. John's Region, Eastern Health **–** Mental Health and Addictions, the NL Centre for Applied Health Research (NLCAHR), the provincial government's Department of Employment and Labour (which is responsible for administrating immigration programs) and, finally, the Association for New Canadians.

**Linkage and exchange** - Approaching and establishing links with the identified research audiences was essential, since the involvement of relevant research stakeholders in the conceptualization of study design and in the mutual sharing of information is the best predictor of consequent research utilization.^1^,^12^ The research models in which research audiences do not participate (like *the knowledge-driven model* or *the science-push model*) have a lower likelihood of adoption and implementation.^13^,^14^ To design my research project according to the *demand-pull model*, I accommodated the ideas and interests of research audiences into my research objectives. For instance, during my pilot study about immigrants’ barriers to utilization of mental health services executed prior to this project, the study participants suggested conducting a larger study examining a variety of social determinants of mental health. Other stakeholders were interested in specific questions related to designing culturally appropriate services and identifying immigrants’ barriers to their utilization. My research audiences, however, not only proposed several key research questions, but also suggested dissemination strategies suitable for their own specific needs, which I address in the section on research communication.

The linkage and exchange phase was important for yet another reason—the interactions with research audiences allow researchers to assess the culture of a receiving environment, an essential factor in determining whether it is conducive to policy change and research implementation. Lomas demonstrated that one of the most important factors to consider is whether the chosen project reflects the values of decision makers: in particular, their interests (its importance to them), beliefs (their assumptions about what is happening) and ideologies (their views about how the world should be).^1^ Indeed, my research audiences reiterated the importance and timeliness of my research since it appeared in the milieu in which the NL Government announced its intention to attract more immigrants in order to stimulate and enhance the economic, social and cultural development of the province struggling with the lowest immigrant arrival and retention rate in Canada.^15^ To be successful, this government initiative intended to design diverse community services that would address the unique needs of local immigrants. This created a window of opportunity for my study examining those immigrants’ needs because it reflected the values of all involved decision makers. In addition to these positive predisposing factors, this new immigration initiative signaled that the provincial government was prepared to allocate significant human, material and financial resources, which are important factors enabling research implementation.^16^

**Research communication** - Increasing awareness of the study is the first essential step toward an effective KT.^17^ However, not all researchers are aware that the traditional one-way communication of research findings in journal publications and scientific conferences (called *diffusion*) does not prove effective in research uptake by policymakers and service providers.^18^,^19^ The other communication modes such as targeted mailing and presentations (called *dissemination*), and interactive workshops (called *implementation*) have much stronger impact on research utilization.^19^ As Choi et al. put it, research “must be actively communicated and marketed” to research audiences who should be actively engaged in developing dissemination and implementation plans.^20^

For this reason, my communication strategies were not limited to journal publications and several presentations at local, national and international conferences but also included three separate seminars: one for policymakers and service providers in immigration, a second for policymakers and service providers in mental health, and a third for researchers, students and faculty conducting research in public health. Study participants attended some of the seminars as well. The communication of my research further included two separate reports for immigration services and health agencies, as well as individual interviews with university counseling services, a local consultancy group, and nursing students on field placement at CMHA. Reports were also made accessible to interested audiences at the university and NLCAHR websites. My reception of a national award for demonstrating excellence in research and KT and the potential impact of my work within the field of health services and policy research sparked the interest of a local radio broadcaster. Discussing my study with callers on a radio show helped in raising awareness about immigrant mental health issues among the general public.

However, dissemination of findings through diverse communication channels *per se* is not a sufficient strategy to increase research utilization. Weiss suggested that communicated messages should be crafted as a narrative rather than a statistical summary.^14^ Moreover, factors such as content filled with actionable messages, a concise appealing format, and the involvement of opinion leaders or “trusted sources” to endorse the research or communicate it directly to decision makers are all very important facilitators of KT.^17^,^21^ Thus, during the research dissemination phase, I focused on providing a compelling package of actionable messages directed at my research audiences while respecting their different communication needs. For instance, while Eastern Health preferred an oral presentation for mental health policymakers and service providers at a local hospital, the government's Immigration Office requested a shorter written report. In addition, involving CMHA and Eastern Health as “trusted sources” proved to be an effective KT strategy, as they promoted my project among other research audiences and provided me with opportunities to further communicate my research.

The last important step in research communication is the follow-up phase during which researchers ascertain whether their recommendations were implemented in order to perform a final evaluation of their KT initiative and to identify the strengths and/or weaknesses of their strategies.^22^ Therefore, I communicated with several research stakeholders after I had disseminated my research to them to find out how my research was used. The outcomes are presented in the following section.

## Results

The implementation of diverse KT strategies surrounding my research project facilitated its utilization among several targeted research audiences. First, my research recommendations were reflected in the province's new immigration strategy.^23^ Furthermore, my research informed the development of the cultural sensitivity training that the Association for New Canadians provided to their employees and enhanced the available immigration and resettlement services. In addition, CMHA released a mental heath promotion brochure entitled “Mental Wellness for Canadian Immigrants” that addressed some of the mental health information needs of the local immigrant community identified in my research. Service providers at the university Student Counseling Centre indicated to me that my findings had effectively informed the services that the Centre offers to international students. Diverse mental health policymakers and care providers indicated that my recommendations were useful in initiating a needed discussion about designing mental health services responsive to the needs of St. John's immigrants. The involvement of diverse stakeholders ensured that health was not discussed in isolation from other important factors such as poverty and discrimination. In addition, my work informed a recent publication of the Canadian Public Health Agency which proposed strategies for addressing a variety of gaps in the area of mental health and illness in Atlantic Canada.^24^

These positive outcomes resulted from implemented KT strategies learned in my KT course. I believe that without a proper environmental assessment, the involvement of research audiences, and effective communication, my research would have been unlikely to reach those policymakers and service providers in a position to implement changes related to the mental health and well-being of immigrants. A good example to support my claim is my previous research on the maternity health and care needs of St. John's immigrant Muslim women, a study that I had conducted before I was exposed to the theory of KT. First, I conducted this study before the provincial government announced its new immigration agenda. Second, I did not establish any links with research audiences. Third, I communicated my findings only through journal publications and conference presentations. As a result, the utilization of this research project by local maternity health policymakers and service providers was minimal. In addition, the success of other research projects utilizing KT precepts documented at a recent Knowledge in Motion International Conference^25^ supports the idea that KT initiatives increase the utilization of research by various research audiences and consumers.

I learned from this experience that publishing research in scientific journals and conferences is helpful for students’ resumes, but initiating a real change requires a broader engagement of the community. In addition, many funding research agencies and policymaking bodies consider KT an important feature of researchers’ grant applications and ask researchers to demonstrate how they will engage users within the research process.^26^ The role of universities in assisting economic growth by providing technologies and knowledge has also been emphasized recently. This role can be effectively achieved only by providing university researchers with skills in KT and outreach.^27^ Moreover, universities that engage in KT can also directly benefit by attracting more resources from industry or funding agencies, and also important (but constantly declining) public support.^28^

All these reasons provide health educators (and others) with a strong rationale for the regular inclusion of KT courses in health sciences curricula. For instance, the American Association of State Colleges and Universities has already responded to these new requirements and expectations by calling for the engagement of post-secondary institutions in “a direct, two-way interaction with communities and other constituencies through the development, exchange and application of knowledge, information and expertise, for mutual benefit.”^29^ Some universities do, in fact, provide students with a variety of opportunities to interact with industry and engage with their communities but, as Bebbington put it, “there are still too many courses where a student can meet all expectations without ever having left their computer screen.”^28^ My KT initiative illustrated that leaving my computer was indeed worthwhile.

## Conclusions

In this paper, I demonstrated one example of the practical application of the KT theory I was exposed to in a KT elective course. I identified the factors that had facilitated the utilization of my research project about the mental health and well-being of St. John's immigrants. These factors can be organized around three main domains of KT: pre-research environmental assessment, linkage and exchange, and post-research communication of findings. During the environmental scan, I assessed the timeliness and relevance of my research and, as well, identified the research audiences with whom I established active links in order to determine their research questions and research communication needs. I also assessed the culture of the receiving environment to ensure that my research would be in accordance with decision makers’ values and to determine that there would be enough human, material and financial resources available in order to facilitate a change. With the help of “trusted sources,” I communicated my research through diverse communication channels, ensuring that the content and format were suitable for the varied needs of the research audiences. Finally, I followed-up on the change implementation outcomes with several stakeholders. All of these strategies proved to be effective in facilitating the utilization of my research.

Equipping students in health disciplines with the knowledge and skills necessary for effective research translation and community outreach will not only enable them to produce research that responds more effectively to the needs of their communities and initiates a change, but it also can be useful in attracting more funding and support from research agencies, industry, government agencies and the public that are increasingly interested in seeing “the benefits of taxpayers dollars that are invested in health research by moving research into policy, programs and practice.”^30^ For all these sound reasons, institutions providing health sciences education should find a standard and well-deserved place in their curricula for KT courses.
